# Early therapy with corticosteroid and surfactant for acute idiopathic pulmonary hemorrhage in infants: Two case reports

**DOI:** 10.1097/MD.0000000000020281

**Published:** 2020-05-22

**Authors:** Yuiko Sato, Mitsutaka Shiota, Kouta Sasaki, Atsuko Hata, Daisuke Hata

**Affiliations:** Department of Pediatrics, Kitano Hospital, Tazuke Kofukai Medical Research Institute, Osaka, Japan.

**Keywords:** acute idiopathic pulmonary hemorrhage, corticosteroid, epistaxis, infant, respiratory distress, surfactant

## Abstract

**Rationale:**

Acute idiopathic pulmonary hemorrhage (AIPH) in infants is a rare condition, and a clear treatment protocol has not yet been established.

**Patient concerns:**

We report 2 infant cases of AIPH in a 3-month-old male and a 1-month-old female, who presented at an emergency room with epistaxis and respiratory distress. Both were immediately intubated, which revealed a bloody intratracheal aspirate.

**Diagnosis:**

Pulmonary hemorrhage was confirmed by X-ray and computed tomography imaging in both cases. The extensive evaluation revealed no specific etiology for the acute pulmonary hemorrhage, and AIPH was therefore diagnosed in both cases.

**Interventions:**

Intravenous methylprednisolone resulted in a rapid improvement in oxygenation and a reduction in high airway pressure during mechanical ventilation. Methylprednisolone was subsequently tapered off within 13 and 3 days in cases 1 and 2, respectively. In case 1, intratracheal administration of a surfactant also resulted in an immediate improvement in respiratory condition and the patient was extubated after 2 days; no effect was seen in case 2, and the patient was extubated after 10 days.

**Outcome:**

Both infants recovered well without sequelae or further relapse after 23 and 71 months of follow-up, respectively.

**Lessons:**

Early administration of corticosteroid therapy and intratracheal administration of diluted surfactant should be considered for severe acute pulmonary hemorrhage in infants.

## 1 Introduction

Acute idiopathic pulmonary hemorrhage (AIPH) in infants is a rare cause of diffuse alveolar hemorrhage. It has sudden onset, causes severe acute respiratory distress in previously healthy infants, and is potentially fatal, suggesting that it may be a cause of sudden infant death syndrome.^[1]^ AIPH is a diagnosis of exclusion, and its diagnosis can be difficult because pediatric pulmonary hemorrhage can result from a variety of causes, including infections, trauma, allergy, bronchiectasis, congenital cardiovascular lesions, immunologic diseases and neoplasms, and all of these causes must be excluded before a definitive diagnosis can be reached.^[2]^ Between January 1993 and November 1994, a total of 10 infants with AIPH was reported in Cleveland, Ohio.^[3,4]^ A search for the cause of AIPH in these infants revealed that pulmonary hemorrhage was associated with household fungi, including the toxin-producing mold Stachybotrys chartarum.^[5]^ However, later investigations failed to prove this association,^[6]^ and the etiology of AIPH is still unclear. Data on incidence is scarce, and most information comes from case reports: 37 cases have been reported in the USA,^[1,3,4,7,8]^ 39 cases in Japan,^[9]^ and 25 and 19 in France^[10]^ and Sweden,^[11]^ respectively. Management is mainly supportive and no specific therapy has yet been established because of the rarity of infant AIPH. AIPH is characterized by the triad of hemoptysis, anemia, and respiratory distress,^[2]^ but since AIPH in infants can manifest as epistaxis, it is important to be alert to the possibility of AIPH in previously healthy patients under 1 year of age who experience a sudden onset of airway bleeding associated with severe respiratory distress. We report 2 cases of AIPH in 2 infants, a 3-month-old male and a 1-month-old female, which emphasize that epistaxis is one of the symptoms of AIPH in infants and early therapeutic intervention with a corticosteroid and a surfactant is critical. Neither patient had a bleeding tendency, but their initial symptom was a nosebleed, which made a differential diagnosis difficult. Both patients recovered after early administration of corticosteroids and in case 1, intratracheal administration of a surfactant also resulted in rapid resolution of respiratory failure.

## 2 Methods

The medical institutional review board of Kitano hospital approved this report.

## 3 Case reports

### 3.1 Case 1

**Figure 1:**
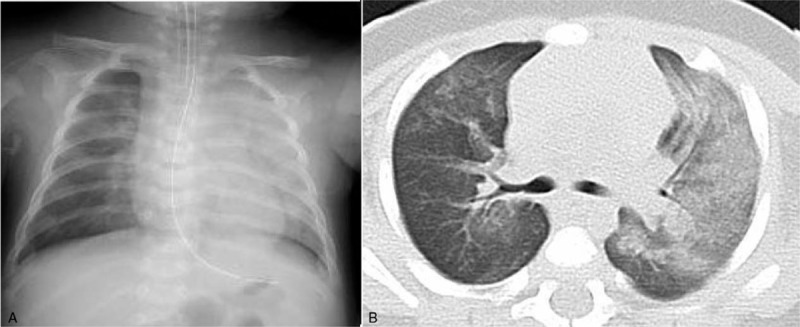
Case 1: (A) Chest radiograph showing diffuse ground glass opacification of the left lung; (B) Computed tomography image showing consolidation in the left upper and lower lobes.

Case 1 was a 3-month-old male admitted with epistaxis and respiratory distress. He was born at 35 weeks’ gestation weighing 2790 g and was admitted to the neonatal intensive care unit for 2 weeks due to transient tachypnea. On physical examination, the patient was febrile (38.3°C) and tachypneic (55 breaths/min) with an oxygen saturation of 75% despite receiving 5 L/min oxygen. He was immediately intubated, and fresh blood was seen in the endotracheal tube. Chest X-ray and computed tomography images were compatible with pulmonary hemorrhage, but no other abnormalities were seen (Fig. 1). Mechanical ventilation at high pressure to achieve a fraction of inspiratory oxygen of 1.0 was initiated but did not improve gas exchange. Intratracheal administrations of 1 mL diluted surfactant (120 mg diluted with 4 mL normal saline) was repeated four times, resulting in a rapid improvement in thoracic movement and hypoxia and a reduced requirement for high airway pressures during ventilation. Intravenous methylprednisolone at a dose of 1 mg/kg every 6 hours was also administered, and the respiratory condition was seen to improve. Worsening anemia was treated with a red blood cell transfusion, and hemoglobin levels decreased from 11.8 g/dL on admission to 8.7 g/dL 2 hours later. As acute respiratory distress in an infant < 4 months old, along with leukocytosis (35,600 /L) as a result of lymphocytosis (65.4%), is suggestive of pertussis, azithromycin treatment was initiated. Although the C-reactive protein level was normal on admission, it increased to 5.35 mg/dL 6 hours later and peaked at 9.0 mg/dL on the next day; fever persisted for 3 days. Therefore, sepsis was considered and cefotaxime and immunoglobulin were administered. Blood culture on admission was negative. Echocardiogram showed mild pulmonary hypertension but no congenital cardiac malformation; coagulation tests were normal. Hematuria and proteinuria detected on admission, in addition to a family history of Churg-Strauss syndrome in his maternal grandfather, suggested autoimmune vasculitis, manifesting as diffuse alveolar hemorrhage. However, the results of urinalysis after intravenous fluid therapy were normal and extensive evaluation identified no specific cause of the pulmonary hemorrhage. Therefore, a diagnosis of infant AIPH was established. The patient's respiratory condition recovered rapidly, resulting in extubation on the third day. His respiratory condition remained stable, and methylprednisolone treatment was tapered off over the following 13 days. He was discharged 14 days after admission, once pulmonary hemosiderosis had been ruled out by an absence of hemosiderin-laden macrophages in the gastric fluid. The patient recovered well and 23 months later had experienced no further sequelae or relapses.

### 3.2 Case 2

**Figure 2:**
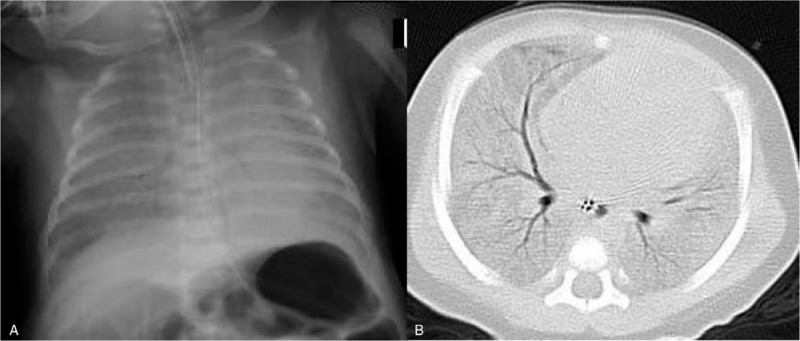
Case 2: (A) Chest radiograph showing widespread ground glass opacification of the bilateral lungs; (B) Computed tomography image showing consolidation with air bronchogram in both lungs.

Case 2 was a 1-month-old female, born at 39 weeks’ gestation weighing 3036 g. She attended the emergency room with epistaxis, although the nosebleed stopped at presentation. She returned home, but 2 hours later, her respiratory condition deteriorated and she returned to the hospital. On physical examination, she was found to be in shock with unmeasurable oxygen saturation and peripheral hypoperfusion and was immediately intubated. The intratracheal aspirate was bloody, and radiologic chest images were compatible with pulmonary hemorrhage (Fig. 2). Despite support with high pressure mechanical ventilation, gas exchange did not improve immediately. Intratracheal administration of surfactant failed to improve the hypoxia or reduce airway pressures. However, intravenous methylprednisolone at a dose of 2 mg/kg every 6 hours resulted in improved oxygenation and a reduction in airway pressure. Methylprednisolone was subsequently tapered to 4 mg/kg/day on the following day and 2 mg/kg/day on the third day, and was discontinued thereafter. Worsening anemia was treated with a red blood cell transfusion on the first day. Echocardiogram images showed no congenital cardiac malformation and an absence of severe pulmonary hypertension. Coagulation tests were normal, and further blood tests revealed no diseases underlying the pulmonary hemorrhage. A diagnosis of infant AIPH was therefore established. The patient was extubated on the tenth day of admission and suffered no further respiratory dyspnea. She recovered well in the following 71 months, with no clinical sequelae or episodes of relapse.

## 4 Discussion

Early treatment with systemic glucocorticoids may be effective in infants with AIPH. Corticosteroids are used empirically in the treatment of many pulmonary conditions, and are administered to most infants with AIPH. In an outbreak of AIPH in the USA, 30 infants were treated for the condition, of whom 25 survived and 23 received corticosteroid therapy, with the authors recommending a methylprednisolone dose of 1 mg/kg every 6 hours.^[8]^ In contrast to the relatively long total period of corticosteroid therapy used in these cases (a mean of 7.8 months), Jacanamijoy et al reported the efficacy of short-course methylprednisolone (4 or 10 days) in 2 cases.^[2]^ As AIPH is so rare, there is currently no conclusive evidence to support the use of corticosteroids in AIPH in infants and it is, therefore, important that all cases are reported to provide cumulative evidence to support the potential benefit of this therapeutic intervention.

The administration of surfactant may also be a promising approach to the treatment of severe respiratory failure with AIPH in infants. In patients with pulmonary hemorrhage, surfactant is inactivated by hemoglobin, red blood cell lipids, and plasma proteins.^[12]^ Therefore, the administration of surfactant aims to reverse these inhibitory effects and the efficacy of this approach has been reported in previous cases.^[13]^ In the current report, case 2 had a longer period after the onset of symptoms and before treatment, meaning that a greater amount of surfactant would have been inactivated; it is therefore likely that more exogenous surfactant may have been required to reverse the inhibitory effect. This may explain the lack of efficacy seen in this case, and we suggest that surfactant should be administered without delay after the onset of pulmonary hemorrhage.

The diagnosis of AIPH in infants can be challenging as pulmonary hemorrhage in children has many different etiologies. As patients with idiopathic pulmonary hemosiderosis require long-term corticosteroid therapy, it is important to exclude this diagnosis. Idiopathic pulmonary hemosiderosis is confirmed by lung biopsy or bronchoscopy with bronchoalveolar lavage (BAL) to detect hemosiderin-laden macrophages, which first appear 3 days after a hemorrhagic episode, with levels peaking at 7 to 10 days and persisting for approximately 2 months in 10% of macrophages.^[14]^ Although it is difficult to perform lung biopsies or bronchoscopy in infants, BAL is available when the patient is intubated. Alternatively, gastric lavage fluid is easily available and the presence of siderophages can provide a simple, but equally diagnostic and reliable, test that can be implemented in all cases.^[15]^

## 5 Conclusions

We describe 2 cases of severe pulmonary hemorrhage leading to intractable hypoxemia. Early administration of corticosteroid therapy and BAL with diluted surfactant resulted in a rapid improvement in gas exchange, suggesting that this approach should be considered for such cases of severe pulmonary hemorrhage.

## Author contributions

**Conceptualization:** Atsuko Hata, Yuiko Sato, Mitsutaka Shiota.

**Data curation:** Yuiko Sato, Kouta Sasaki.

**Supervision:** Mitsutaka Shiota, Daisuke Hata.

**Validation:** Yuiko Sato.

**Writing – original draft:** Yuiko Sato.

**Writing – review & editing:** Mitsutaka Shiota, Daisuke Hata.
